# NK cell activity and methylated *HOXA9* ctDNA as prognostic biomarkers in patients with non-small cell lung cancer treated with PD-1/PD-L1 inhibitors

**DOI:** 10.1038/s41416-023-02285-z

**Published:** 2023-05-03

**Authors:** Sara Witting Christensen Wen, Line Nederby, Rikke Fredslund Andersen, Torben Schjødt Hansen, Christa Haugaard Nyhus, Ole Hilberg, Anders Jakobsen, Torben Frøstrup Hansen

**Affiliations:** 1 Department of Oncology, Vejle Hospital, University Hospital of Southern Denmark, Beriderbakken 4, 7100 Vejle, Denmark; 2 Department of Regional Health Research, University of Southern Denmark, J.B. Winsloews Vej 19, 3rd floor, 5000 Odense C, Denmark; 3 Department of Biochemistry and Immunology, Vejle Hospital, University Hospital of Southern Denmark, Beriderbakken 4, 7100 Vejle, Denmark; 4 Department of Medicine, Vejle Hospital, University Hospital of Southern Denmark, Beriderbakken 4, 7100 Vejle, Denmark

**Keywords:** Prognostic markers, Non-small-cell lung cancer, DNA, Innate immune cells, Immunotherapy

## Abstract

**Background:**

PD-1/PD-L1 inhibitors have improved survival for patients with non-small cell lung cancer (NSCLC). We evaluated natural killer cell activity (NKA) and methylated *HOXA9* circulating tumor DNA (ctDNA) as prognostic biomarkers in NSCLC patients treated with PD-1/PD-L1 inhibitors.

**Methods:**

Plasma was prospectively collected from 71 NSCLC patients before treatment with PD-1/PD-L1 inhibitors and before cycles 2–4. We used the NK Vue^®^ assay to measure the level of interferon gamma (IFNγ) as a surrogate for NKA. Methylated *HOXA9* was measured by droplet digital PCR.

**Results:**

A score combining NKA and ctDNA status measured after one treatment cycle had a strong prognostic impact. Group 1 had IFNγ < 250 pg/ml and detectable ctDNA (*n* = 27), group 2 consisted of patients with either low levels of IFNγ and undetectable ctDNA or high levels of IFNγ and detectable ctDNA (*n* = 29), group 3 had IFNγ ≥250 pg/ml and undetectable ctDNA (*n* = 15). Median OS was 221 days (95% CI 121–539 days), 419 days (95% CI 235–650 days), and 1158 days (95% CI 250 days—not reached), respectively (*P* = 0.002). Group 1 had a poor prognosis with a hazard ratio of 5.560 (95% CI 2.359–13.101, *n* = 71, *P* < 0.001) adjusting for PD-L1 status, histology, and performance status.

**Conclusions:**

Combining NKA and ctDNA status after one cycle of treatment was prognostic in patients with NSCLC treated with PD-1/PD-L1 inhibitors.

## Background

Immunotherapy has greatly improved the survival and supplied an alternative to chemotherapy for patients with advanced non-small cell lung cancer (NSCLC) with tumor programmed death ligand-1 (PD-L1) expression as low as 1% [1–3] or even irrespective of PD-L1 expression [4].

Currently, tissue PD-L1 is the only biomarker routinely used in treatment with programmed death-1 (PD-1)/PD-L1 inhibitor therapy [5–8]. PD-L1 plays an important role in helping cancer cells avoid detection and elimination by the immune system [9, 10]. Only about 20% of patients have a long-term survival benefit from this treatment, and some patients experience treatment response and long overall survival (OS) despite a low tissue PD-L1 expression [2, 11, 12] demonstrating the complex interaction between the tumor microenvironment and the host immune system. Hence, there is a need for biomarkers with reliable prognostic value for the individual patient treated with PD-1/PD-L1 inhibitors.

Natural Killer (NK) cells are known for the ability to target and kill infected or malignant cells without previous antigen presentation [13–15]. They also play an important role in activating other immune cells and they secrete cytokines and chemokines that both orchestrate other elements of the immune system and directly affect transformed and infected cells [16–19]. Previously, tumor infiltrating NK cells have been found to be of prognostic value when investigated by immunohistochemistry in lung cancer biopsies [20–22] and currently, great efforts are being made to augment the NK cell’s activity towards tumors [23], with a recent study demonstrating improved response rate and progression-free survival in pembrolizumab-treated NSCLC patients treated with autologous expanded NK cells [24]. In recent years, an assay that artificially activates the NK cells in peripheral blood and then measures the level of interferon gamma (IFNγ) as a readout for NK cell response has opened the possibility of estimating the level of NK cell activity (NKA) in whole blood without isolating and culturing the cells [25–27]. It measures the level of IFNγ produced in the blood collection tube as a surrogate marker for NKA. By use of this assay NKA has shown prognostic value in ovarian cancer [28], prostate cancer [28], colorectal cancer [28], gastric cancer [29], and lung cancer [30, 31].

Cell-free circulating tumor DNA (ctDNA) has been suggested as a prognostic biomarker in multiple solid tumor types treated with immune check-point inhibitors [32] or chemotherapy [33]. The ctDNA dynamics during treatment have been proposed for early response evaluation [32, 34–36]. We have shown that detectable ctDNA in patients with Stage III–IV NSCLC receiving first-line chemotherapy or targeted treatment was an indicator of poor prognosis using methylated homeobox A9 (*HOXA9*) as biomarker [37]. *HOXA9* is a gene encoding a transcription factor, but the exact function is not yet known. It is known to play a role in haematopoiesis and in the development of some haematologic cancers such as acute myeloid leukemia [38–40]. Its downregulation has been shown to augment the migratory potential of lung cancer cell lines [41, 42].

The host immune system plays a pivotal role in the effectiveness of PD-1/PD-L1 inhibitor treatment, and ctDNA has shown promise as a marker of early response. Hence, a combination of immunologic markers and ctDNA markers may be superior to either type of marker alone as suggested by Nabet and colleagues [36]. We aimed to evaluate NKA and methylation of the *HOXA9* promoter region as prognostic biomarkers in patients with advanced non-small cell lung cancer treated with PD-1/PD-L1 inhibitors.

## Materials and methods

### Study population

Patients were prospectively recruited per protocol at Vejle Hospital, Department of Oncology, between September 2017 and November 2019. Patients were followed until December 2021. Inclusion criteria comprised age >18 years and monotherapy treatment with PD-1/PD-L1 inhibitor for NSCLC in any line, while the primary exclusion criteria were actionable mutations and other experimental treatment within 14 days. Performance status was evaluated using the Eastern Cooperative Oncology Group performance status classification. NSCLC was staged according to tumor node metastasis (TNM) 8th edition and PD-L1 tumor expression assessed using immunohistochemistry (PD-L1 IHC 22C3 pharmDx, DAKO Agilent, Santa Clara, California, USA). All patients gave written, informed consent. The study was performed in accordance with the Declaration of Helsinki and approved by the Regional Committee on Health Research Ethics for Southern Denmark (S-20170063).

### Treatment and evaluation

All patients were treated and followed according to national Danish guidelines. They received pembrolizumab or atezolizumab intravenously every three weeks at the recommended individual doses. Nivolumab was administered intravenously every two weeks. Treatment was evaluated with chest and abdominal computed tomography (CT) scans every three treatment cycles. Response to treatment was assessed according to RECIST 1.1 criteria [43]. Disease progression was based on a clinical evaluation in the cases where RECIST 1.1. assessment was not possible. Treatment continued until disease progression or until two full years of treatment. Response evaluation was performed blinded to the biomarker status of the patient. Study endpoints were progression-free survival (PFS) defined as number of days from treatment cycle 2 until disease progression or death, and overall survival (OS) defined as number of days from treatment cycle 2 to death. Patients were censored at data work-up on December 17, 2021, if they had not experienced progression or death.

### Study samples

Blood was sampled at baseline and before each subsequent treatment cycle. Nine ml venous blood was sampled in an EDTA containing tube for DNA analysis. The tube was centrifuged at 2000×*g* for 10 min, plasma was collected and stored at –80 °C until analysis. Storage time ranged from 6 to 30 months. One ml venous blood was sampled into a Promoca containing NK Vue® tube [44] for analysis of NKA and placed in an incubator at 37 °C within 15 min of sampling. After 20–24 h of incubation the plasma was collected and stored at –80 °C until analysis. Maximum storage time was 1 month.

### NK cell activity

The level of IFNγ as a surrogate for NKA was measured using the NK Vue® Kit (NKMAX, Seongnam-si, South Korea). The method has previously been reported by our group [26, 28]. The frozen plasma samples were thawed at room temperature and centrifuged at 11,500×*g* for 1 min before they were analyzed using the NK Vue® enzyme-linked immunosorbent assay (ELISA) (NKMAX, Seongnam-si, South Korea). All steps were carried out according to the manufacturer’s instructions. Test results above the assay’s upper limit of quantification (2000 pg/ml) were reanalyzed in a 1:10 dilution. Three serum samples with low, intermediate and high level of IFNγ were included as controls in each run. Measured in-house, the intra-assay coefficient of variation of the ELISA was <10% while the inter-assay coefficient of variation was <12%. A level of <250 pg/ml IFNγ was considered abnormal as per the manufacturer’s instructions. The analysis was performed blinded to the clinical endpoints.

### Circulating methylated *HOXA9*

The method was previously published by our group [45]. Briefly, 4 ml plasma was thawed at room temperature and centrifuged at 10,000×*g* for 10 min. DNA was extracted using the DSP Circulating DNA kit (Qiagen, Hilden, Germany) as per the manufacturer’s instructions. The DNA extraction process was quality controlled by adding CPP1 [46], a non-human gene, while the total amount of cell-free DNA was estimated using the β2 microglobulin gene. The purified DNA was bisulfite converted using the EZ DNA Methylation-Lightning Kit (Zymo Research, Irvine, California, USA) following the manufacturer’s instructions. The converted DNA was analyzed for methylation of the promoter region of the *HOXA9* gene using an in-house droplet digital polymerase chain reaction (ddPCR) assay [45]. The primer and probe sequences as well as the PCR settings are available in the Supplementary Materials in Section 1 and Supplementary Table S1. Water was used as negative control, lymphocyte DNA from healthy donors (20 µl corresponding to ~20 ng) as non-cancer control, and Universal Methylated DNA Standard (Zymo Research, Irvine, California, USA) as positive control. All controls were analyzed in parallel with the patient samples. We used a QX200 Automated Droplet Generator (Bio-Rad, Hercules, California, USA) for generating droplets and a QX200 Droplet Digital Reader (Bio-Rad, Hercules, California, USA) for reading the results. A limit of detection was set at 5 droplets containing methylated *HOXA9* as previously reported [45]. Data were then normalized to the level of the albumin gene and presented as a percentage of methylated *HOXA9* including a 95% confidence interval [47]. Methylated *HOXA9* was considered undetectable if the value was 0% or if the 95% confidence interval included 0. The analysis was performed blinded to the clinical endpoints.

### Power calculation

The study was planned as an explorative study, since NKA is not yet thoroughly investigated. We estimated that the response rate would be 30% for the whole cohort. Dividing the patients according to NKA, we assumed response rates of 20% and 40%, respectively, for low NKA and high NKA. With a risk of Type 1 error of 5% and a power of 80%, we would be able to distinguish a statistically significant difference with a cohort of 80 patients.

### Statistical analyses

Numeric variables were presented as median and interquartile range, while categorical variables were presented as number and percentage. We used the Fisher’s exact test for group comparisons. We used the Kruskal–Wallis test for comparing numeric values between groups, while the Kruskal Wallis multiple comparison Z-value test was used for comparisons between repeated measurements. Kaplan–Meier statistics and Logrank test were used to evaluate the association between biomarkers and clinical endpoints. Unadjusted hazard ratios (HR) were calculated by simple Cox regression. A multiple Cox regression model was created using the backward selection method. More information about the multiple Cox regression model can be found in the Supplementary Materials. Two-sided *P* values < 0.05 were considered significant. All analyses except Cox regression were performed using NCSS 11 (NCSS, Utah, USA). Cox regression modelling was performed using STATA 17BE (StataCorp LLC, Texas, USA).

## Results

### Patient selection and characteristics

Initially, 83 patients met the inclusion and exclusion criteria for the protocol “NK Check”, but one patient was later discovered to have a *ROS1* gene rearrangement and was excluded. Supplementary Fig. S1 shows the number of patients included and analyzed at each step of the study. At baseline, 80 and 78 of the patients were analyzed for methylated *HOXA9* and NKA, respectively. The patient characteristics for all 82 patients and divided according to baseline biomarker status can be viewed in Supplementary Table S2. After the first cycle of treatment, 71 patients were available for the combined biomarker analysis.

Patient characteristics are shown in Table 1 for the whole cohort and divided according to the combined biomarker score. This cohort had a high frequency of adenocarcinomas, primarily advanced-stage cancer, very few never-smokers, and a high degree of PD-L1 expression.

**Table 1:** Patient characteristics.

Patient characteristics	Total *n* = 71	Group 1, *n* = 27	Group 2, *n* = 29	Group 3, *n* = 15	*P* value
Sex, male	33 (46%)	13 (48%)	14 (48%)	6 (40%)	0.907
Age, years	70 (66–5)	69 (66–4)	69 (67–4)	75 (66–9)	0.211
Histologic type					0.659
Adenocarcinoma	49 (69%)	21 (78%)	18 (62%)	10 (67%)	
Squamous cell carcinoma	15 (21%)	5 (19%)	7 (24%)	3 (20%)	
Other^a^	7 (10%)	1 (4%)	4 (14%)	2 (13%)	
Stage					0.140
III	5 (7%)	0 (0%)	5 (17%)	0 (0%)	
IV	56 (79%)	23 (85%)	20 (69%)	13 (87%)	
Unknown^b^	10 (14%)	4 (15%)	4 (14%)	2 (13%)	
Treatment					0.146
Pembrolizumab	67 (94%)	24 (89%)	29 (100%)	14 (93%)	
Atezolizumab	2 (3%)	1 (4%)	0 (0%)	1 (7%)	
Nivolumab	2 (3%)	2 (7%)	0 (0%)	0 (0%)	
Previous lines of treatment					0.271
0	48 (68%)	15 (56%)	22 (76%)	11 (73%)	
1	21 (30%)	11 (41%)	7 (24%)	3 (20%)	
2	2 (3%)	1 (4%)	0 (0%)	1 (7%)	
Smoking status					0.191
Never	2 (3%)	1 (4%)	0 (0%)	1 (7%)	
Former	47 (66%)	16 (59%)	18 (62%)	13 (87%)	
Active	19 (27%)	8 (30%)	10 (34%)	1 (7%)	
Unknown^b^	3 (4%)	2 (7%)	1 (3%)	0 (0%)	
Performance status					0.844
0	38 (54%)	15 (56%)	14 (48%)	9 (60%)	
1	29 (41%)	10 (37%)	14 (48%)	5 (33%)	
2	4 (6%)	2 (7%)	1 (3%)	1 (7%)	
PD-L1 expression					0.096
<50%	16 (23%)	9 (33%)	3 (10%)	4 (27%)	
≥50%	55 (77%)	18 (67%)	26 (90%)	11 (73%)	

Patient characteristics for the 71 patients included in the combined biomarker score. Group 1 had an abnormal level of IFNγ (<250 pg/ml) and detectable methylated *HOXA9* (*n* = 27), group 2 had either abnormal levels of IFNγ and undetectable methylated *HOXA9* or normal levels of IFNγ and detectable methylated *HOXA9* (*n* = 29), and group 3 had normal levels of IFNγ (≥250 pg/ml) and undetectable methylated *HOXA9* (*n* = 15). Results are presented as median and interquartile range (IQR) for numeric variables and as a number and percentage (%) for categorical variables. Percentages may not add up to 100 due to rounding. The *P* values represent Fisher’s exact test for categorical data and Kruskal Wallis test for numeric data.

^a^This category comprises poorly differentiated non-small cell lung cancer, large cell lung carcinoma, and pulmonary sarcomatoid carcinoma.

^b^No data regarding stage or smoking status in the medical record.

### NK cell activity and methylated *HOXA9*

The level of IFNγ varied greatly across the cohort. The median values were 158 pg/ml (range 0–6466 pg/ml), 154 pg/ml (range 0–10,420 pg/ml), and 217 pg/ml (range 0–5862 pg/ml) at baseline, before cycle 2, and before cycle 4, respectively. There was no difference between the values at baseline and before the second or fourth cycle (*P* = 0.497). At baseline, 48/78 (61.5%) patients had abnormally low levels of IFNγ (<250 pg/ml), while this fraction was 46/74 (62.2%) before the second cycle and 31/59 (52.5%) before the fourth treatment cycle.

**Fig. 1:**
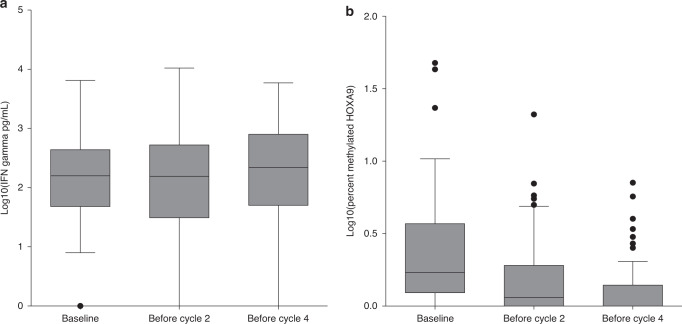
Biomarker levels of NK cell activity and aberrant methylation of the *HOXA9* promoter region. **a** Box plot illustrating the log10 transformed levels of interferon gamma (IFNγ) in pg/ml at baseline, before cycle 2, and before cycle 4, immediately after control CT scan. **b** Box plot illustrating the log10 transformed levels of methylated HOXA9 circulating tumor DNA in percent at the same time points as Fig. 1a.

The median levels of methylated *HOXA9* were 0.7% (range 0–46.6%), 0.2% (range 0–20%), and 0% (range 0–6.1%) at baseline, before cycle 2, and before cycle 4, respectively. There was a statistically significant difference between the values at baseline and before the second and fourth cycle (*P* < 0.001). At baseline, 66/80 (82.5%) patients had detectable methylated *HOXA9*. The fraction was 41/73 (56.2%) before the second cycle and 22/56 (39.3%) before the fourth treatment cycle. The values were log10-transformed for better visual accessibility in Fig. 1 (for untransformed values see Supplementary Fig. S2).

### Prognostic impact of dynamic NK cell activity

**Fig. 2:**
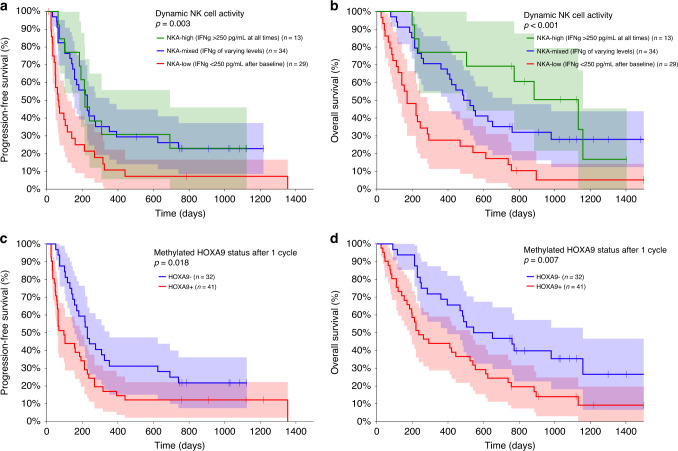
Prognostic impact of dynamic NK cell activity and methylated *HOXA9* status. Impact of NK cell activity (NKA) on progression-free survival (**a**, *P* = 0.003) and overall survival (**b**, *P* < 0.001). The red line represents the group of patients (NKA-low, *n* = 29) with an abnormally low level of IFNγ or who dropped to and remained at a low level (<250 pg/ml) at all available time points. The blue line represents the group of patients (NKA-mixed, *n* = 34) who experienced either an increase from abnormal to normal levels of IFNγ or vice versa. The green line represents the patients (NKA-high, *n* = 13) who maintained normal levels of IFNγ (≥250 pg/ml) at all available time points. Impact of methylated *HOXA9* status after one cycle of treatment on progression-free survival (**c**, *P* = 0.018) and overall survival (**d**, *P* = 0.007). The red line represents the group of patients (*HOXA9* + , *n* = 41) with detectable methylated *HOXA9*, while blue line represents the group of patients (*HOXA9*–, *n* = 32) with no detectable methylated *HOXA9*. Shaded areas represent the 95% confidence intervals.

We classified the patients into three groups according to dynamic NKA as inspired by a previous report [28]. We based the classification on the results of four time-points; baseline, before cycle 2, before cycle 3, and before cycle 4, immediately after the first CT evaluation. If the patient stopped treatment before all four samples were taken, the classification was based on the samples available. The NKA-low group had a persistently low level of IFNγ or dropped to and remained at a low level after baseline (<250 pg/ml, *n* = 29), the NKA-mixed group experienced either an increase from abnormal to normal levels or vice versa (*n* = 34), while the NKA-high group maintained a normal level of IFNγ (≥250 pg/ml, *n* = 13). There was a strong relationship between dynamic NKA and both PFS (Fig. 2a) and OS (Fig. 2b). The median PFS was 64 days (95% CI 48–115 days), 228 days (95% CI 146–353 days), and 214 days (95% CI 101–693 days), respectively, for NKA-low, NKA-mixed, and NKA-high (*P* = 0.003). The median OS was 170 days (95% CI 110–285 days), 487 days (95% CI 361–761 days), and 1131 days (95% CI 235 days to not reached), respectively, for NKA-low, NKA-mixed and NKA-high (*P* < 0.001). There was no statistically significant difference at baseline for either PFS (*P* = 0.152) or OS (*P* = 0.062, see Supplementary Fig. S3), nor if the median value at baseline (158 pg/ml) was used as cut-off (PFS *P* = 0.455, OS *P* = 0.088, Supplementary Fig. S4).

### Prognostic impact of methylated *HOXA9* status

We divided the patients according to methylated *HOXA9* status as previously reported [37]. The *HOXA9* + group had detectable methylated *HOXA9* after one cycle of treatment (*n* = 41), while the *HOXA9*- group had no detectable methylated *HOXA9* (*n* = 32). There was a statistically significant association between methylated *HOXA9* status and both PFS (Fig. 2c) and OS (Fig. 2d). Median PFS was 97 days (95% CI 58–192 days) and 228 days (95% CI 146–353 days), respectively, for *HOXA9* + and *HOXA9*– (*P* = 0.018). Median OS was 235 days (95% CI 170–525 days) and 544 days (95% CI 361–1158 days), respectively, for *HOXA9* + and *HOXA9*– (*P* = 0.007). There was no statistically significant difference at baseline for neither PFS (*P* = 0.275) nor OS (*P* = 0.059, Supplementary Fig. S5).

### Prognostic impact of a combined biomarker score

**Fig. 3:**
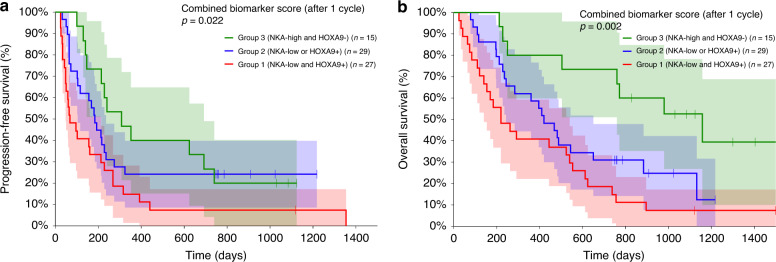
Prognostic impact of a score combining NK cell activity (NKA) and methylated *HOXA9* after the first treatment cycle. There was a statistically significant impact on both progression-free survival (**a**, *P* = 0.022) and overall survival (**b**, *P* = 0.002). The red line represents the group of patients (group 1, *n* = 27) with IFNγ < 250 pg/ml and detectable levels of methylated *HOXA9*. The blue line represents the group of patients (group 2, *n* = 29) with either low levels of IFNγ and undetectable methylated *HOXA9* or high levels of IFNγ and detectable methylated *HOXA9*. The green line represents the patients (group 3, *n* = 15) with a high level (≥250 pg/ml) of IFNγ and undetectable methylated *HOXA9*. Shaded areas represent the 95% confidence intervals.

We calculated a combined biomarker score classifying patients according to their biomarker status at a single time-point after the first treatment cycle. Group 1 had an abnormal level of IFNγ (<250 pg/ml) and detectable methylated *HOXA9* (*n* = 27), group 2 had either abnormal levels of IFNγ and undetectable methylated *HOXA9* or normal levels of IFNγ and detectable methylated *HOXA9* (*n* = 29), and group 3 had normal levels of IFNγ (≥250 pg/ml) and undetectable methylated *HOXA9* (n = 15). We found a clear association between the biomarker score and both PFS (Fig. 3a) and OS (Fig. 3b). Median PFS was 69 days (95% CI 48–213 days), 183 days (95% CI 102–235 days), and 307 days (95% CI 140–693 days), respectively, for group 1, 2, and 3 (*P* = 0.022). Median OS was 221 days (95% CI 121–539 days), 419 days (95% CI 235–650 days), and 1158 days (95% CI 250 days to not reached), respectively, for group 1, 2, and 3 (*P* = 0.002). There was a statistically significant impact of the combined biomarker score at baseline for OS (*P* = 0.049) but not for PFS (*P* = 0.187, Supplementary Fig. S6).

A total of 66 patients had CT scans evaluable by RECIST 1.1 after three cycles of treatment. Response to treatment was grouped as non-progression (partial response and stable disease, there were no patients with complete response) and progression (progressive disease). The non-progression rates were 55%, 76%, and 93%, respectively, for group 1, 2, and 3. There was a statistically significant difference in response rates between groups (*P* = 0.029).

Both dynamic NKA groups and methylated *HOXA9* status were statistically significant prognostic factors in simple and multiple Cox regression analyses (see Supplementary Tables S4–S6). Biomarker score 1 (abnormal level of IFNγ < 250 pg/ml and detectable methylated *HOXA9* after the first treatment cycle) was a marker of poor prognosis with a HR of 3.971 (95% CI 1.763–8.943, *P* = 0.001) compared to biomarker score 3 (normal IFNγ ≥250 pg/ml and undetectable methylated *HOXA9*, Table 2). It remained statistically significant with a HR of 5.560 (95% CI 2.359–13.101, *P* < 0.001) when adjusting for PD-L1 status, histology, and performance status (Table 2). Further information about the multiple Cox regression model can be viewed in Supplementary Materials Table S3.

**Table 2:** Multiple Cox regression model.

	Unadjusted			Adjusted		
Variable	HR	95% CI	*P*	HR	95% CI	*P*
Biomarker score, 3	1			1		
2	2.435	1.070–5.540	0.034	2.211	0.951–5.138	0.065
1	3.971	1.763–8.943	0.001	5.560	2.359–13.101	<0.001
PDL1 status, <50%	1			1		
≥50%	1.325	0.696–2.523	0.391	2.791	1.206–6.458	0.017
Histology, adenocarcinoma	1			1		
Other^a^	1.621	0.950–2.766	0.077	1.991	1.034–3.832	0.039
Performance status, 0	1			1		
1–2	1.833	1.087–3.091	0.023	2.219	1.230–4.006	0.008

Multiple Cox regression analysis performed on 71 patients with 55 deaths occurring during the observation time. The group with biomarker score 1 had an abnormal level of IFNγ (<250 pg/ml) and detectable methylated *HOXA9*, group 2 had either abnormal levels of IFNγ and undetectable methylated *HOXA9* or normal levels of IFNγ and detectable methylated *HOXA9*, and group 3 had normal levels of IFNγ (≥250 pg/ml) and undetectable methylated *HOXA9*. Unadjusted values represent simple Cox regression analyses. Adjusted values represent the full multiple Cox regression model.

^a^This category comprises primarily squamous cell carcinoma. Please refer to footnote of Table 1 for the full list of other histologic types included.

## Discussion

Immunotherapy with PD-1/PD-L1 inhibitors has revolutionized the treatment of NSCLC, but there is a need for better prognostic, but also predictive, biomarkers. In the present study we show that dynamic measures of IFNγ as a surrogate for NKA and ctDNA in the form of methylated *HOXA9* are prognostic factors associated with both PFS and OS. A combined biomarker score enhances the prognostic impact, which is retained in a multiple Cox regression analysis.

The immune system has been widely investigated in relation to PD-1/PD-L1 inhibitor immunotherapy. Backman and colleagues [22] found that a subgroup of NSCLC patients with an NK cell and/or plasma cell pattern of infiltration had a favorable prognosis even though CD8 + T cell infiltration was low. Tumor infiltration with NK cells was also found to be a favorable prognostic marker in previous studies [20, 21]. However, functional cell analysis has also shown promise as a prognostic biomarker [29, 31]. Conventionally, NKA has been accounted for by measuring the cytotoxic potential of the cells. This has been done by a ^51^Cr-release assay involving loading of living target cells with radioactive chromium and then measuring the level of ^51^Cr in the supernatant as a surrogate for cell death [48]. Alternatively, NK cell cytotoxicity can be assessed by flow cytometry [26, 49]. Although equally fit for measuring NKA, both techniques are time- and labor-intensive. This makes them excellent research tools, however implementing them as standard assays in high-throughput clinical laboratories is challenging.

As an alternative to the traditional cytotoxicity assays, we used the NK Vue^®^ assay that measures IFNγ levels after stimulation with an NK cell activator as a surrogate for NKA [26]. Utilizing this method, we show here that classification according to dynamic NKA was strongly associated with prognosis in our cohort of patients with NSCLC treated with PD-1/PD-L1 inhibitors. This is in line with findings by Choi and colleagues [31], who reported significantly longer PFS in the NSCLC group with high NKA at baseline compared to the NSCLC group with low activity. In the Choi study, they used a cut-off of 1200 pg/ml IFNγ based on receiver operating characteristic (ROC) analysis of their dataset (using non-progressors as the reference) and not the standard cut-off of 250 pg/ml recommended by the manufacturer. Another strategy is to use the median value of IFNγ as cut-off as was done by Cho and colleagues [29] in a gastric cancer cohort, and by Lee and colleagues [50] in a pancreatic ductal adenocarcinoma cohort. In our data, there was no statistically significant association between single time-point NKA at baseline and PFS or OS regardless of whether we used the median NKA value or 250 pg/ml as cut-off.

A number of differences between the present study and the work by Choi and colleagues [31] may contribute to the differences observed. Specifically, we analyzed more than twice as many patients for dynamic NKA, and in our cohort the percentage of never smokers was much smaller and the fraction of patients with PD-L1 < 50% was much higher than was reported by Choi and colleagues. Furthermore, while almost half the patients in the Choi cohort were treated with a PD-L1 inhibitor, this was only the case for about 5% of patients in this report. Our group has previously reported a study [28] in patients with stage 4 ovarian-, prostate-, or colorectal cancer. They were grouped according to the dynamics of NKA, as we did in this study, and the group with abnormally low dynamic NKA had significantly lower response rates and shorter PFS. This was also demonstrated by Cho and colleagues [29] in HER2 + advanced gastric cancer patients treated with first-line fluoropyrimidine–platinum doublet plus trastuzumab, and by Lee and colleagues [50] in pancreatic ductal adenocarcinoma treated with conventional chemotherapy, where low dynamic NKA was associated with worse PFS and OS. This underscores the importance of a functioning immune system regardless of cancer type and treatment.

In a previous study, our group reported a strong prognostic impact of ctDNA in the form of methylated *HOXA9* in patients with advanced NSCLC receiving first-line treatment [37]. We therefore investigated whether methylated *HOXA9* may be associated with the prognosis of patients treated with PD-1/PD-L1 inhibitors. We found that both PFS and OS were more than doubled in the patients who had no detectable methylated *HOXA9* compared to the patients with detectable levels after the first cycle of treatment. These findings are supported by Goldberg and colleagues [35] who investigated 28 patients with NSCLC receiving PD-1/PD-L1 inhibitors and found that a decrease in ctDNA >50% from baseline was associated with a better prognosis. Similar results were reported by Raja and colleagues [34], who discovered that a decrease in variant allele frequency between baseline and 6 weeks of treatment was related to radiographic response and survival in NSCLC patients treated with durvalumab. Their results were validated in an independent cohort.

In the studies by Goldberg and colleagues [35] and Raja and colleagues [34], ctDNA was measured by next-generation sequencing (NGS) targeting 24 and 73 genes, respectively, while our study employed methylation-specific ddPCR of only one gene, *HOXA9*. Still, we detected methylated *HOXA9* in 82% of the patient samples at baseline indicating that this is a useful common ctDNA marker. The study by Goldberg and colleagues [35] only detected ctDNA in 57% of the baseline plasma samples. Conversely, 93% and 90% of NSCLC patients in the Discovery and Validation cohorts, respectively, had detectable ctDNA at baseline in the study by Raja and colleagues [34]. This may reflect the larger gene panel used, since both studies extracted DNA from 1 ml of plasma and had a similar composition of disease stages. We did not validate our findings in an independent cohort, but we did find that 78% of 231 patients with stage III and IV NSCLC had detectable methylated *HOXA9* before initiating treatment in a previous study [37].

Nabet and colleagues [36] developed a biomarker score in order to predict durable clinical benefit of PD-1/PD-L1 inhibitors. A Bayesian multiparameter model comprising normalized blood tumor mutational burden, CD8 + T cell fraction, and ctDNA dynamics predicted durable clinical benefit with 92% accuracy in both the Discovery and Validation cohorts, and patients with a favorable score had significantly longer PFS. Theoretically, it makes good sense to integrate tumor factors like ctDNA and host factors like immune function in order to increase the performance of a biomarker. We present a score combining NKA and ctDNA, which shows a clear association with both PFS and OS. Only best score vs. worst score retained statistical significance in a multiple Cox regression model, whereas the intermediate score did not reach statistical significance. This may be due to the relatively small number of patients in each group.

The primary limitations of the present study are the relatively modest cohort size of 71 patients evaluable with the combined biomarker score and the lack of a validation cohort. We might have split the cohort into a Discovery and a Validation cohort, but our results should be interpreted with caution because some of the small biomarker groups contain only 13 or 15 patients. Our cohort was too small to include all possible clinical variables in the multiple regression model, but we did include the most relevant prognostic variables, PD-L1 status, histology, and performance status. Age and sex were not found to be of prognostic value in this model and were therefore excluded.

The study design was a prospective collection of samples for analysis of NKA and ctDNA, but the ctDNA analyses were performed retrospectively. The sample storage time ranged from 6-30 months, which increases the risk of cell-free DNA degradation. However, we used a percentage of ctDNA normalized to the level of the circulating, cell-free albumin gene instead of the absolute copy number of methylated *HOXA9*. This should reduce the effect a potential DNA loss would have on the results.

The NK Vue^®^ test was developed within the past ten years and is a relatively new method for estimating NKA. It measures IFNγ after incubation with a stimulant as a surrogate for NKA. Since other cells like T cells and natural killer T cells also secrete IFNγ, the level of IFNγ does not directly translate to the level of NKA. However, a previous study showed that the fraction of IFNγ-producing NK cells by far exceeded the other cell types [26]. Therefore, we believe this test to be a valuable alternative to the conventional methods and more suitable for routine clinical practice.

In conclusion, a biomarker score combining the levels of NKA and methylated *HOXA9* after the first cycle of treatment can be used to stratify the prognosis of patients with NSCLC treated with PD-1/PD-L1 inhibitors. The two methods, NK Vue^®^ and methylation-specific ddPCR, are quick, inexpensive, and could easily be implemented into routine clinical practice. Future research is needed to validate these findings in a larger cohort.

## Supplementary information

Supplementary materials

## Data Availability

The data presented in this study are available on request from the corresponding author. The data are not publicly available due to ethical considerations.
