# Supplementary material for: NK cell activity and methylated HOXA9 ctDNA as prognostic biomarkers in patients with non-small cell lung cancer treated with PD-1/PD-L1 inhibitors
Source: Br J Cancer. 2023 May 3;129(1):135–42. doi: 10.1038/s41416-023-02285-z (PMC10307873; doi:10.1038/s41416-023-02285-z)
Supplement: Supplementary file 1 — Supplementary materials [file 41416_2023_2285_MOESM1_ESM.pdf]

NK cell activity and methylated HOXA9 ctDNA as prognostic biomarkers in patients with non-small cell lung cancer treated with PD-1/PD-L1 inhibitors

Supplementary materials

1. Methods

Methylated HOXA9 measured by ddPCR

Methylated HOXA9 was analyzed with an in-house ddPCR assay (primers and probe from LGC Biosearch technologies, Aarhus, Denmark).

Primer sequence	
Forward	GAGTATTTCGATTTTAGTTCGTGT
Reverse	CGCGTACACTAAATTCCAC

Probe sequence	
Probe	FAM-TTAGTTTAAGGCGACGGTGTT-BHQ-1

Positive control:
Universal Methylated Human DNA Standard, 2 µL (DNA concentration 250 ng/µL, Zymo Research, Irvine, California, USA), healthy donor lymphocyte DNA 199 µL (DNA concentration approximately 20 ng/µL) and water 800 µL.
For each reaction, 20 µL of this mixture was added and bisulfite converted in parallel with the patient samples.

Table S1: PCR conditions for the methylation specific droplet digital PCR assay

Steps	Temperature	Time
Step 1	95ºC	10 minutes
Step 2: 44 cycles	95ºC<br>56ºC	15 seconds<br>1 minute
Step 3	98ºC	10 minutes

Machine: Veriti Thermal Cycler (Applied Biosystems, Thermo Fisher Scientific, Foster City, California, USA).
Ramp rate: 1.5ºC/second.

PD-L1 expression measured by immunohistochemistry
Analysis for tumor cell PD-L1 expression was routinely performed on formalin fixed paraffin embedded biopsies from the diagnostic workup. The PD-L1 IHC 22C3 pharmDx (DAKO Agilent, Santa Clara, California, USA) was used according to the manufacturer's instructions.

2. Patient selection and characteristics

Figure S1: Patients included and analyzed at each step of the study

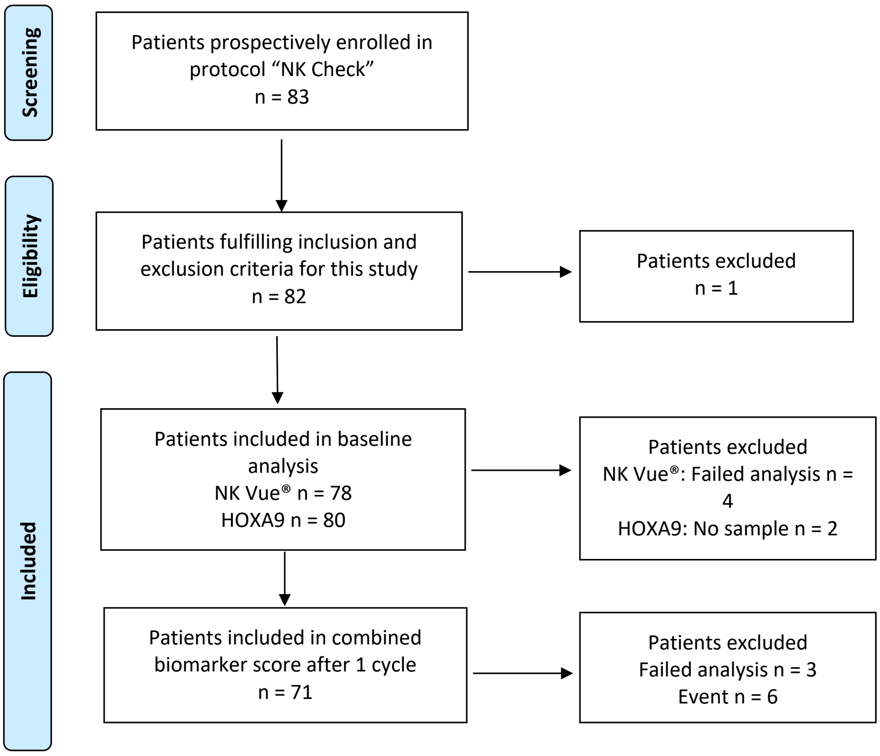

Figure S1: Flowchart illustrating patient selection and inclusion for analysis. One patient fulfilled inclusion and exclusion criteria but turned out to have a targetable ROS1 gene rearrangement and was therefore excluded.

Table S2: Patient characteristics for the whole cohort and divided according to biomarker status

Patient characteristics	Total n = 82	HOXA9- n = 32	HOXA+ n = 41	NKA-low n = 29	NKA-mixed n = 34	NKA-high n = 13
Sex, male	35 (43%)	16 (50%)	18 (44%)	14 (48%)	14 (41%)	6 (46%)
Age, years	70 (66-75)	71 (66-76)	69 (67-74)	70 (66-73)	69 (66-75)	73 (68-78)
Histologic type						
Adenocarcinoma	58 (71%)	20 (63%)	29 (71%)	22 (76%)	21 (62%)	9 (69%)
Squamous cell carcinoma	17 (21%)	8 (25%)	9 (22%)	5 (17%)	9 (26%)	3 (23%)
Other[1]	7 (9%)	4 (13%)	3 (7%)	2 (7%)	4 (12%)	1 (8%)
Stage						
II	2 (2%)	0 (0%)	1 (2%)	1 (3%)	0 (0%)	0 (0%)
III	6 (7%)	3 (9%)	2 (5%)	2 (7%)	3 (9%)	1 (8%)
IV	63 (77%)	23 (72%)	33 (80%)	22 (76%)	26 (76%)	10 (77%)
Missing	11 (13%)	6 (19%)	5 (12%)	4 (14%)	5 (15%)	2 (15%)
Treatment						
Pembrolizumab	76 (93%)	30 (94%)	38 (93%)	28 (97%)	31 (91%)	12 (92%)
Atezolizumab	3 (4%)	2 (6%)	1 (2%)	1 (3%)	1 (3%)	1 (8%)
Nivolumab	3 (4%)	0 (0%)	2 (5%)	0 (0%)	2 (6%)	0 (0%)
Previous lines of treatment						
0	55 (67%)	22 (69%)	27 (66%)	16 (55%)	26 (76%)	9 (69%)
1	25 (30%)	9 (28%)	13 (32%)	12 (41%)	8 (24%)	3 (23%)
2	2 (2%)	1 (3%)	1 (2%)	1 (3%)	0 (0%)	1 (8%)
Smoking status						
Never	2 (2%)	1 (3%)	1 (2%)	1 (3%)	0 (0%)	1 (8%)
Former	56 (68%)	23 (72%)	25 (61%)	14 (48%)	25 (74%)	11 (85%)
Active	21 (26%)	7 (22%)	13 (32%)	11 (38%)	9 (26%)	1 (8%)
Unknown[2]	3 (4%)	1 (3%)	2 (5%)	3 (10%)	0 (0%)	0 (0%)
Performance status						
0	45 (55%)	16 (50%)	23 (56%)	15 (52%)	20 (59%)	7 (54%)
1	33 (40%)	15 (47%)	15 (37%)	12 (41%)	12 (35%)	6 (46%)
2	4 (5%)	1 (3%)	3 (7%)	2 (7%)	2 (6%)	0 (0%)
PD-L1 expression						
<50%	17 (21%)	6 (19%)	11 (27%)	7 (24%)	5 (15%)	5 (38%)
≥50%	65 (79%)	26 (81%)	30 (73%)	22 (76%)	29 (85%)	8 (62%)

Table S2: Patient characteristics for the 82 included patients and divided according to biomarker status after the first cycle of treatment. Results are presented as median and interquartile range (IQR) for numeric variables and as a number and percentage (%) for categorical variables. Percentages may not add up to 100 due to rounding. [1]This category comprises poorly differentiated non-small cell lung cancer, large cell lung carcinoma, and pulmonary sarcomatoid carcinoma. [2]No data regarding smoking status in the medical record.

3. Biomarker levels

Figure S2: Biomarker levels

A B

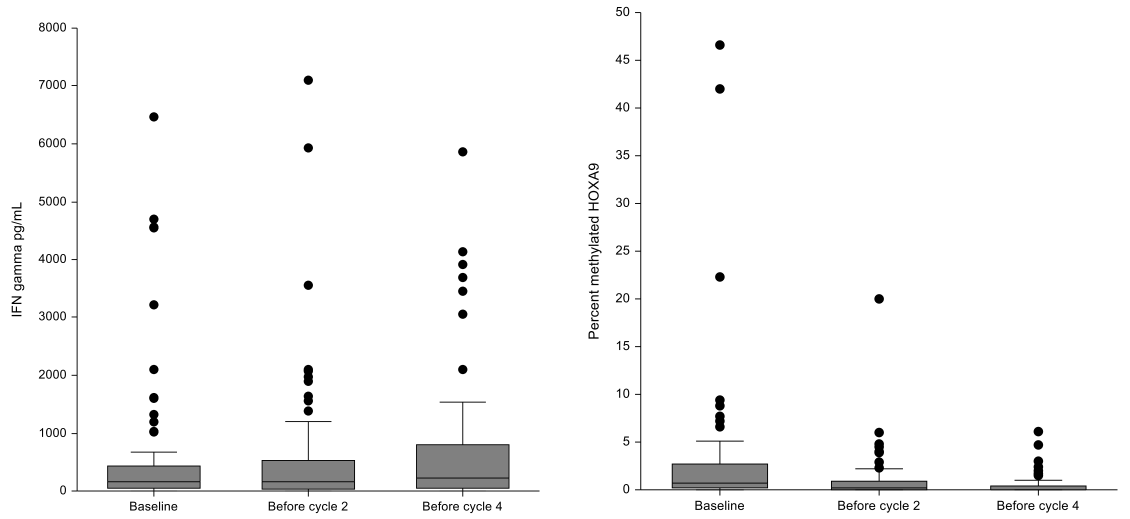

Figure S2: The levels of IFNγ (A) and methylated HOXA9 (B) at baseline, after the first treatment cycle, and after the third treatment cycle. IFNγ was measured in pg/mL and methylated HOXA9 as a percentage normalized to the Albumin gene.

3. Kaplan-Meier analysis

Figure S3: NK cell activity at baseline

A B

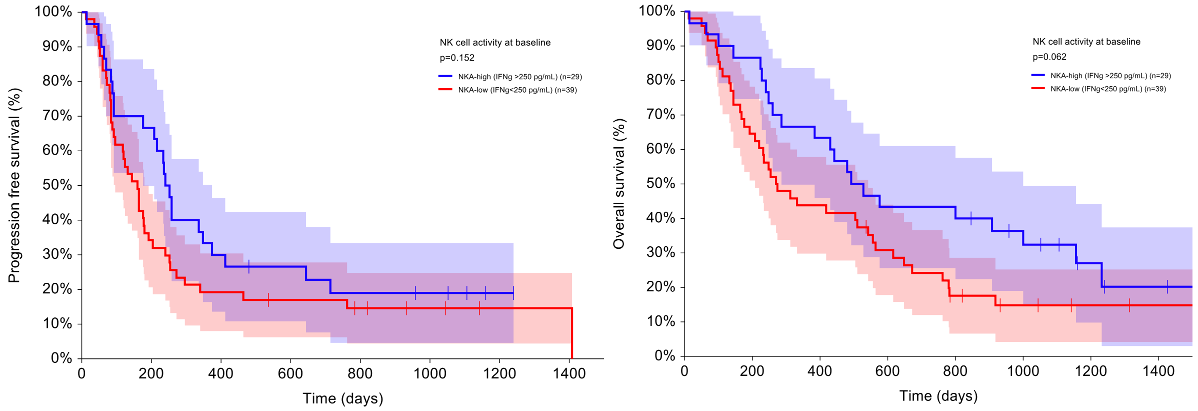

Figure S3: Prognostic impact of NK cell activity at baseline on progression free survival (A, p=0.152) and overall survival (B, p=0.062). The red line represents the group of patients (n=39) who had abnormal levels of IFNγ (<250 pg/mL) at baseline. The blue line represents the patients (n=29) who had normal levels of IFNγ (≥250 pg/mL) at baseline.

Figure S4: NK cell activity at baseline, median value at baseline used as cut-off

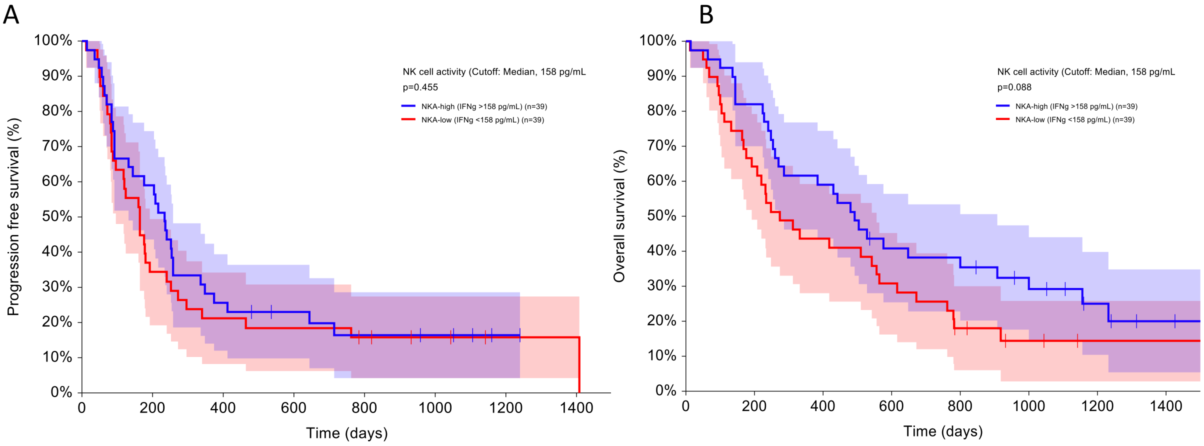

Figure S4: Prognostic impact of NK cell activity at baseline on progression free survival (A, p=0.455) and overall survival (B, p=0.088). The median value at baseline was used as a cut-off to distinguish high vs. low NK cell activity. The red line represents the group of patients (n=39) who had lower levels of IFNγ (<158 pg/mL) at baseline. The blue line represents the patients (n=39) who had higher levels of IFNγ ($\geq$158 pg/mL) at baseline.

Figure S5: Methylated HOXA9 at baseline

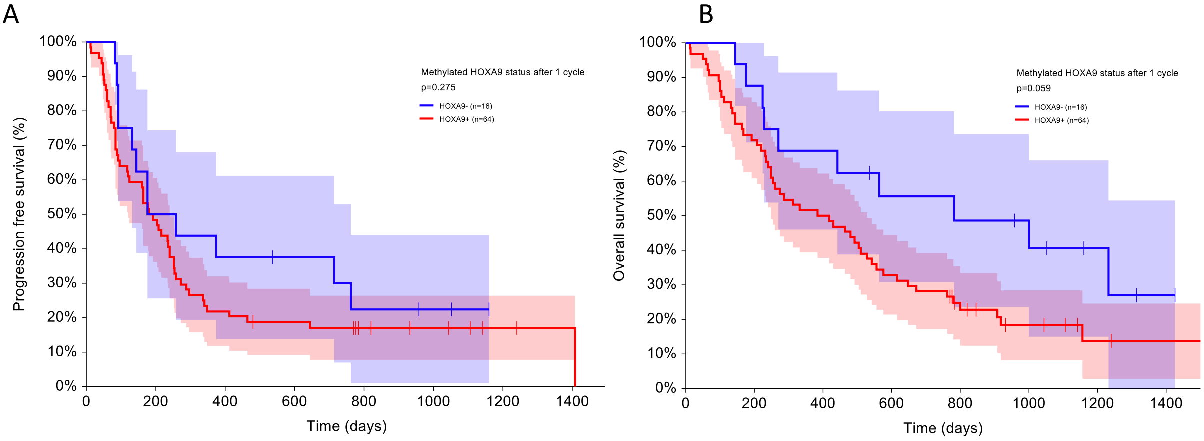

Figure S5: Prognostic impact of methylated HOXA9 status at baseline on progression free survival (A, p=0.275) and overall survival (B, p=0.059). The red line represents the group of patients (HOXA9+, n=64) with detectable methylated HOXA9, while the blue line represents the group of patients (HOXA9-, n=16) with no detectable methylated HOXA9.

Figure S6: Combined biomarker score at baseline

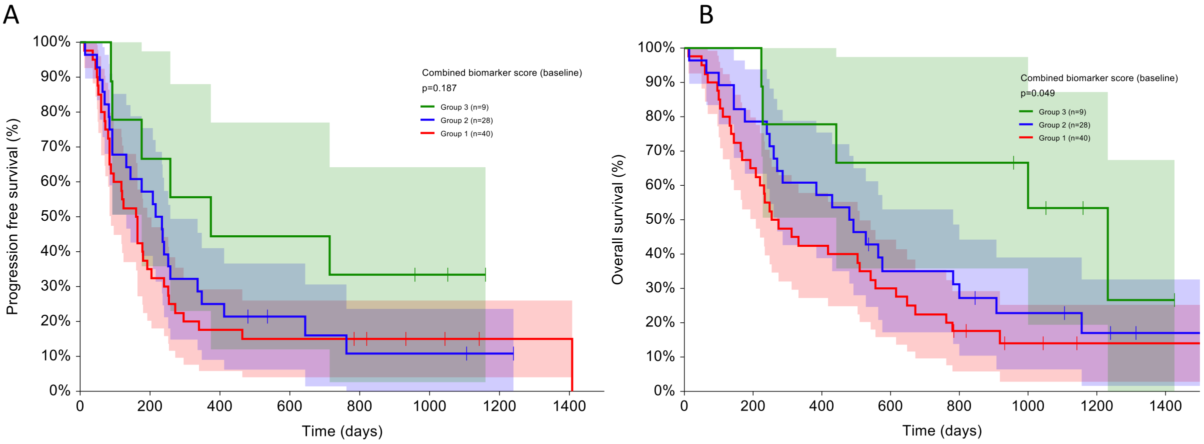

Figure S6: Prognostic impact of a score combining NKA and methylated HOXA9 at baseline on progression free survival (A, p=0.187) and overall survival (B, p=0.049). The red line represents the group of patients (group 1, n=40) with IFNγ < 250 pg/mL and detectable levels of methylated HOXA9. The blue line represents the group of patients (group 2, n=28) with either low levels of IFNγ and undetectable methylated HOXA9 or high levels of IFNγ and detectable methylated HOXA9. The green line represents the patients (group 3, n=9) with a high level (≥250 pg/mL) of IFNγ and undetectable methylated HOXA9. Shaded areas represent the 95% confidence intervals.

Figure S5: PD-L1 status

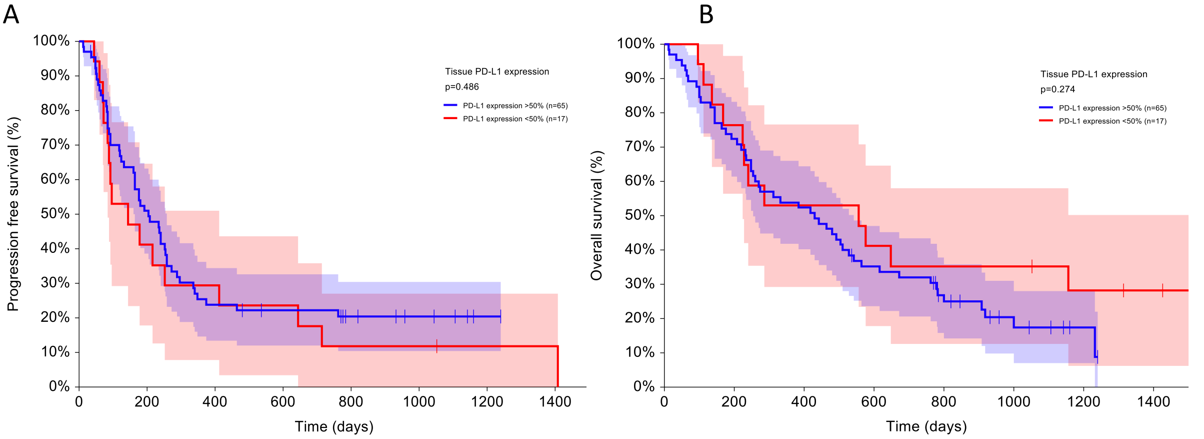

Figure S5: Prognostic impact of PD-L1 status on progression free survival (A, p=0.486) and overall survival (B, p=0.274). The red line represents the group of patients (n=65) with PD-L1 expression ≥50%, while the blue line represents the group of patients (n=17) with PD-L1 expression <50%.

Figure S6: Histology

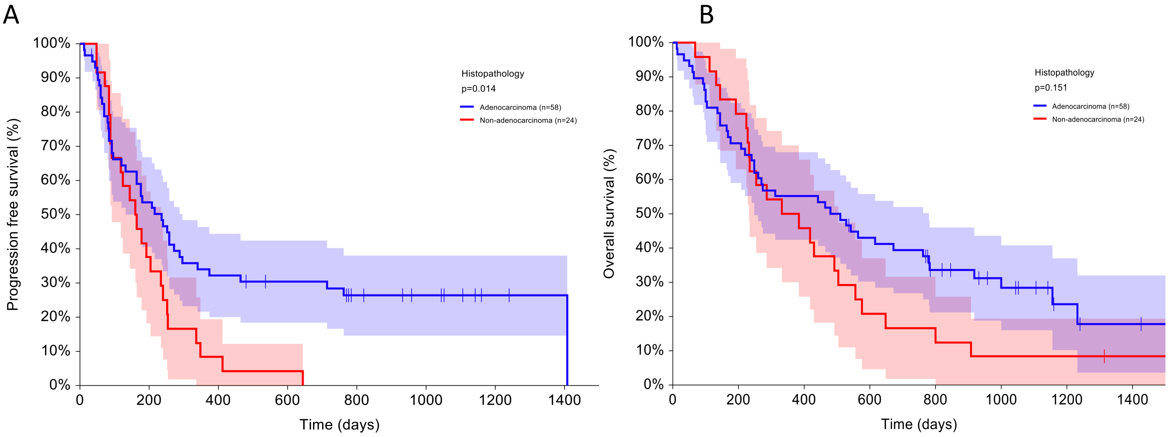

Figure S6: Prognostic impact of histology on progression free survival (A, p=0.014) and overall survival (B, p=0.151). The blue line represents the group of patients (n=58) with adenocarcinoma, while the red line represents the group of patients (n=24) with other histologic cancer types. This category comprises primarily squamous cell carcinoma. Please refer to Table 1 for the full list of other histologic types included.

4. Cox regression analysis

The multiple Cox regression model was developed using the backward selection method. The model initially comprised biomarker score, PD-L1 status, histology, age, and sex. Backward selection was performed by fitting the model with and without the covariate with the highest p-value comparing the larger model with the smaller (nested) model by the Likelihood-ratio test. If there was no significant difference between the two models, the missing covariate did not contribute significantly to the model and the smaller model was kept for further model development.

Table S3: Detailed multiple Cox regression model

Cox regression with no ties
No. of subjects = 71 Number of obs = 71
No. of failures = 55
Time at risk = 38,094 (days) LR chi2(5) = 28.15
Log likelihood = -186.80685 Prob > chi2 = 0.0000

Variable	HR	Std.err.	z	p-value	95% CI lower	95% CI upper
Biomarker score, 3	1					
2	2.135688	0.9202595	1.76	0.078	0.9178221	4.969551
1	4.761171	2.027098	3.67	<0.001	2.066852	10.96777
PDL1 status, <50%	1					
≥50%	2.288115	0.9254773	2.05	0.041	1.035598	5.055506
Histology, adenocarcinoma	1					
Other[1]	2.318227	0.7543038	2.58	0.010	1.225159	4.386515
Performance status, 0	1					
1-2	2.219412	0.6686358	2.65	0.008	1.229699	4.005686

Table S3: Multiple Cox regression analysis performed on 71 patients with 55 deaths occurring during the observation time. The group with biomarker score 1 had an abnormal level of IFNγ (<250 pg/mL) and detectable methylated HOXA9, group 2 had either abnormal levels of IFNγ and undetectable methylated HOXA9 or normal levels of IFNγ and detectable methylated HOXA9, and group 3 had normal levels of IFNγ (≥250 pg/mL) and undetectable methylated HOXA9. [1]This category comprises primarily squamous cell carcinoma. Please refer to Table 1 for the full list of other histologic types included.

Both methylated HOXA9 status after the first treatment cycle and NK cell activity classified as NKA-high, NKA-mixed, and NKA-low (see Results) were of significant prognostic impact (Supplementary Table S4). They were also independent prognostic factors in a multiple model (Supplementary Table S5).

Table S4: Simple Cox regression analysis

Variable	HR	Std.err.	z	p-value	95% CI lower	95% CI upper
HOXA9 status, detectable	2.079313	0.5741393	2.65	0.008	1.210281	3.572345
NKA status, abnormal	2.119515	0 .617154	2.58	0.010	1.197806	3.75048
NKA-high	1					
NKA-mixed	2.486404	0.114	3.21	0.001	1.425102	4.338084
NKA-low	3.689859	0.111	3.20	0.001	1.656833	8.217514
Biomarker score, 3	1					
2	2.435011	1.021198	2.12	0.034	1.070343	5.539607
1	3.970619	1.644857	3.33	0.001	1.762958	8.942818
PD-L1 status, high	1.325195	0.435384	0.86	0.391	0.6960225	2.523111
Histology, other	1.620861	0.4420152	1.77	0.077	0.949773	2.766125
Performance status, 1-2	1.833406	0.488576	2.27	0.023	1.087492	3.090947

Table S4: Simple Cox regression analysis of the variables included in the final multiple model (Table 2 and Table S3) and the alternative multiple models (Table S5 and Table S6).

Table S5: Multiple Cox regression model including dynamic NK cell activity and methylated HOXA9 status

Cox regression with no ties
No. of subjects = 73 Number of obs = 73
No. of failures = 57
Time at risk = 38,352 (days) LR chi2(5) = 25.07
Log likelihood = -196.94754 Prob > chi2 = 0.0001

Variable	HR	Std.err.	z	p-value	95% CI lower	95% CI upper
HOXA9 status, undetectable	1					
Detectable	1.894313	0.5522054	2.19	0.028	1.069844	3.354153
NKA-high	1					
NKA-mixed	2.205828	0.1362377	2.63	0.008	1.223971	3.975319
NKA-low	3.170930	0.1338529	2.72	0.007	1.380080	7.285661
PDL1 status, <50%	1					
>50%	2.061507	0.7400099	2.02	0.044	1.020077	4.166168
Histology, adenocarcinoma	1					
Other[1]	2.503496	0.7683785	2.99	0.003	1.371811	4.568773

Table S5: Multiple Cox regression analysis performed on 73 patients with 57 deaths occurring during the observation time. Performance status was not included in this model, as the data can only support five variables (55 deaths occurring). [1]This category comprises primarily squamous cell carcinoma. Please refer to the footnote of Table 1 for the full list of other histologic types included.

Table S6: Multiple Cox regression model including NK cell activity and methylated HOXA9 status both measured after the first treatment cycle

Cox regression with no ties
No. of subjects = 71 Number of obs = 71
No. of failures = 55
Time at risk = 38,094 (days) LR chi2(5) = 28.45
Log likelihood = -186.65412 Prob > chi2 = 0.0000

Variable	HR	Std.err.	z	p-value	95% CI lower	95% CI upper
HOXA9 status, undetectable	1					
Detectable	2.719081	0.836415	3.25	0.001	1.487937	4.968894
NKA status, normal	1					
NKA status, abnormal	2.096696	0.6362939	2.44	0.015	1.156691	3.800611
PDL1 status, <50%	1					
>50%	2.789919	1.136592	2.52	0.012	1.255505	6.199616
Histology, adenocarcinoma	1					
Other[1]	1.982535	0.6524723	2.08	0.038	1.040116	3.778852
Performance status, 0	1					
1-2	2.293341	07020819	2.71	0.007	1.258585	4.178832

Table S6: Multiple Cox regression analysis performed on 71 patients with 55 deaths occurring during the observation time. [1]This category comprises primarily squamous cell carcinoma. Please refer to the footnote of Table 1 for the full list of other histologic types included.
